# Innate Orientating Behavior of a Multi-Legged Robot Driven by the Neural Circuits of *C. elegans*

**DOI:** 10.3390/biomimetics9060314

**Published:** 2024-05-23

**Authors:** Kangxin Hu, Yu Zhang, Fei Ding, Dun Yang, Yang Yu, Ying Yu, Qingyun Wang, Hexi Baoyin

**Affiliations:** 1 School of Aeronautic Science and Engineering, Beihang University, Beijing 100191, China; hukangxin@buaa.edu.cn (K.H.); m1805331752@163.com (F.D.); dunyang@buaa.edu.cn (D.Y.); yuyingmath@163.com (Y.Y.); nmqingyun@163.com (Q.W.); 2 School of Aerospace Engineering, Tsinghua University, Beijing 100084, China; zhangyu23@mails.tsinghua.edu.cn (Y.Z.); baoyin@tsinghua.edu.cn (H.B.)

**Keywords:** biological neural network, *C. elegans*, neural dynamics, computing model of whole-brain neural network, bionic robot intelligent control

## Abstract

The objective of this research is to achieve biologically autonomous control by utilizing a whole-brain network model, drawing inspiration from biological neural networks to enhance the development of bionic intelligence. Here, we constructed a whole-brain neural network model of Caenorhabditis elegans (*C. elegans*), which characterizes the electrochemical processes at the level of the cellular synapses. The neural network simulation integrates computational programming and the visualization of the neurons and synapse connections of *C. elegans*, containing the specific controllable circuits and their dynamic characteristics. To illustrate the biological neural network (BNN)’s particular intelligent control capability, we introduced an innovative methodology for applying the BNN model to a 12-legged robot’s movement control. Two methods were designed, one involving orientation control and the other involving locomotion generation, to demonstrate the intelligent control performance of the BNN. Both the simulation and experimental results indicate that the robot exhibits more autonomy and a more intelligent movement performance under BNN control. The systematic approach of employing the whole-brain BNN for robot control provides biomimetic research with a framework that has been substantiated by innovative methodologies and validated through the observed positive outcomes. This method is established as follows: (1) two integrated dynamic models of the *C. elegans*’ whole-brain network and the robot moving dynamics are built, and all of the controllable circuits are discovered and verified; (2) real-time communication is achieved between the BNN model and the robot’s dynamical model, both in the simulation and the experiments, including applicable encoding and decoding algorithms, facilitating their collaborative operation; (3) the designed mechanisms using the BNN model to control the robot are shown to be effective through numerical and experimental tests, focusing on ‘foraging’ behavior control and locomotion control.

## 1. Introduction

A biological neural network (BNN) is the core brain that controls a creature’s intelligent behaviors through a unique network structure and integrative control principles. It also inspires the control of human-designed autonomous agents, including robots; more advanced intelligent behaviors can be generated by mimicking the neural control mechanism of creatures with a BNN at a deeper and more interactive level. In recent years, researchers have increasingly focused on digitizing a living brain [1,2,3] due to the new progress in the use of the BNN to investigate biological intelligence, including its dynamic properties and the control mechanisms behind its specific behaviors. Since the BNN possesses complex and efficient dynamic properties to realize biological intelligence, it is valuable to study its intrinsic network structure and dynamical mechanisms within the context of robotics control applications [4]. This endeavor aims to derive insights from biological intelligence, thereby facilitating the development of more advanced robotic behaviors through the emulation of BNN functionalities. According to the experimental data, most parts of the whole-brain neural network system of *C. elegans*, including the micro-level and control functions, have been exploited [5], which is the reason why a systematic model of this biological neuron network was built through programming. A whole-brain dynamical neural computing simulation was completed, and the whole-brain network was visualized, with color projection imaging corresponding to its electrical responses, to validate the entire model and its controllable circuits.

To achieve the goal of robot control through a BNN, there has been lots of research on brain-inspired intelligent control and its applications in recent years. The research conducted by Mathias et al. [6] utilized a liquid neuron network inspired by the neural structure of *C. elegans*. This network consisted of only 19 neurons and was used to enable autonomous driving in both cars and drones. This technology is primarily used for controlling parts of the whole car’s driving system but is limited to relatively simple driving scenarios. In the OpenWorm project [7], some researchers put a whole-brain model of the biological neuron network of *C. elegans* into a Lego robot [8]. Their work involved controlling the robot to avoid obstacles, for example, turning to avoid colliding with a wall. However, it did not realize more complex behaviors, as its obstacle-avoidance performance was not sufficient. Thomas et al. [9] developed a robot with worm-like motion that also utilizes the biological neural network of *C. elegans* for control. This robot is able to exhibit similar behaviors to *C. elegans*, but has certain limitations in terms of its appearance and physical structure. Similarly, Deng et al. simulated part of the biological neural circuit of *C. elegans* and built a simulation environment for a worm-like robot mechanism [10]; however, this approach neglected whole-brain model simulation and did not include real experiments. These studies developed a feasible methodology for replicating the complex behaviors and neural networks of *C. elegans* in robot systems but highlighted the challenges and limitations faced in systematic verification and experiments. Consequently, we decided to tackle these issues by completing a whole-brain model and implementing appropriate and persuasive control methods for both simulation and experiments.

Here, we present a systematic method to make the BNN system and robot kinetic system interact with each other to control a robot’s movement through intelligent behavior instead of mimicking the movement of the worm. The intelligent behaviors of *C. elegans*, like foraging and obstacle avoidance, are consistent with the requirements for controlling a field robot [11], so a form of robot control based on similar intelligent behaviors can be inspired by the BNN’s unique characteristics when controlling *C. elegans*. The foraging and locomotion behaviors of *C. elegans* are exclusively controlled by specific circuits within the network and are referred to in previous experiments [12,13,14,15,16] (a locomotion control circuit in [12,14,16], mechanosensory circuits in [13], and a navigation circuit in [15]). We discovered and verified all the controllable circuits by analyzing the model’s simulation outputs. The designed 12-legged radial skeleton robot possesses the function of walking on complex terrain [17] with a completely developed kinetic model. In order to integrate the network as the control component of this robotic system, we designed pragmatic control mechanisms and methodologies to implement the BNN control module to regulate the robot’s motion. This encompassed the establishment of encoding and decoding protocols facilitating the exchange of variables between the BNN system and the kinetic framework of the robot. These two systems can communicate with each other in real time to control the robot through inter-process communication [18] while using two independent programming environments. The results show that BNN control could enable the robot to move in an autonomous and intelligent way using this efficient and unique control programming in both simulation and experiments.

This paper is organized as follows.

In Section 2, detailed modeling methods are discussed, including the simulation of the whole-brain BNN, the robot’s kinetics, and the experimental platform. In Section 3, the BNN model is illustrated, the design process of combining the BNN system with the robot kinetic system to enable the robot to make intelligent movements for specific missions is elaborated, and the simulation and experiment results are presented. In Section 4, the research contributions are summarized, along with an analysis.

## 2. Materials and Methods

### 2.1. Dynamic Simulation of C. elegans’ BNN

#### 2.1.1. The Whole-Brain Structure of *C. elegans*

**Figure 1:**
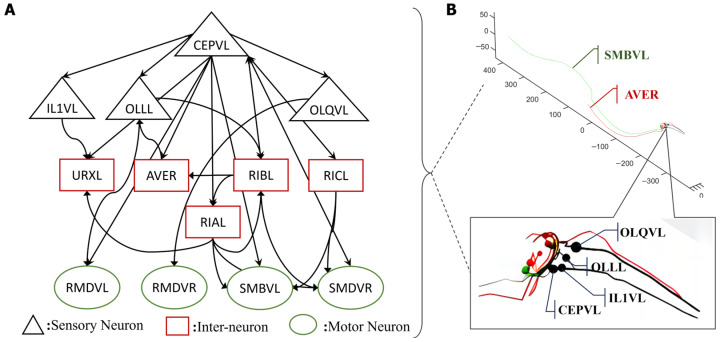
One critical circuit containing sensory neuron CEPVL and the relevant parts of the interneurons and motor neurons. In the visualization diagram of (**B**), the detailed positions of sensory neuron CEPVL, interneuron AVER and motor neuron SMBVL are highlighted, as well as their connections, which are shown in the first illustration with different colors representing the different types of neurons, consistent with (**A**). In (**A**), every node represents a single neuron. The sensory neuron CEPVL senses the stimuli from the head and then propagates them to the interneurons that are distributed around it and the motor neurons distributed along the body to drive muscles to complete the corresponding movement, consistent with the morphological structure in (**B**). The synapse connection is shown in (**A**), expressed by an arrow. It describes the whole connection, from several sensory neurons, signified by black triangles, to the interneurons, signified by red rectangles, and the motor neurons, signified by green ellipses.

The entire nervous system of an adult hermaphrodite *C. elegans* contains 302 neurons and over 5000 synapses based on the original anatomy data [16,19]. Its intelligent behaviors of undulatory motion, turning, and obstacle avoidance are determined by its distributed neuron networks and muscles. The biological neuron network of *C. elegans* can be divided into three distinct layers that perform specific functions: sensory neurons, interneurons, and motor neurons. The role of sensory neurons is to convert a specific type of stimuli into action potentials through their receptors, known as sensory transduction. Typically, these stimuli originate from nature, such as temperature fluctuations [20] or food concentration changes [21]. Additionally, sensory neurons in *C. elegans* are capable of mediating responses to physical stimuli, including harsh mechanical forces [22]. The research demonstrates that *C. elegans* possesses specialized mechanosensory neurons, including neurons called ASH [23,24], OLQ [24,25], CEP [26], and ADE [22,24], which sense specific mechanical stimuli. Furthermore, different sensory neurons manipulate various types of stimuli [22]. Upon activation, these neurons receive converted current input of a distinct type [22] which, in turn, generates action potentials via specific ion channels included in the neural network structure. Secondly, interneurons enable communication between the sensory and motor neurons, and serve as central nodes for the transmission of electrical impulses in neural circuits. Thirdly, motor neurons stimulate muscle fiber by receiving impulses from presynaptic neurons, and finally, diverse motor neurons control various reactive movements of *C. elegans* through synapse connections with muscles. For instance, DA [27] motor neurons control backward locomotion, and DB [28] motor neurons regulate forward locomotion. The motor neurons RMD, RMB, SMB, and SMD [29] regulate head-turning movements. The overall structure consists of three layers of neurons that form multiple functional circuits for controlling *C. elegans* and the whole neural network. For example, Figure 1 shows the circuit, comprising the sensory neuron CEPVL, interneurons, and motor neurons, along with their topological connections. The synapse connection relationship between neurons and the related data, including their polarity and weights, were all extracted from the EleganSign project website [30].

#### 2.1.2. Modelling the Neural Network of *C. elegans*

In terms of the network’s fundamental structure and properties, building a model of the whole BNN of *C. elegans* involves constructing individual neurons and their synaptic connections. Certain mathematical theorems establish the dynamic properties derived from both components. The model follows the Hodgkin–Huxley (HH) rule [31] for an individual neuron, which describes the relationship between its membrane potential and current, in line with the fundamentals of neuroscience. A set of nonlinear differential equations indicates that the current or voltage response can be numerically simulated through programming.

(1) $$\{\begin{array}{c} I=C_{m}\frac{dV_{m}}{dt}+{\bar{g}}_{K}n^{4}\left(V_{m}-V_{K}\right)+{\bar{g}}_{Na}m^{3}h\left(V_{m}-V_{Na}\right)+{\bar{g}}_{l}\left(V_{m}-V_{l}\right)\\ \frac{dn}{dt}=\alpha_{n}\left(V_{m}\right)\left(1-n\right)-\beta_{n}\left(V_{m}\right)n\\ \frac{dm}{dt}=\alpha_{m}\left(V_{m}\right)\left(1-m\right)-\beta_{m}\left(V_{m}\right)m\\ \frac{dh}{dt}=\alpha_{h}\left(V_{m}\right)\left(1-h\right)-\beta_{h}\left(V_{m}\right)h \end{array}$$

where I refers to the current applied to neurons, $V_{m}$ represents the membrane potential, and $C_{m}$ denotes the electrical conductance of a neuron membrane. This has a constant value for all 302 neurons in this model. Furthermore, ${\bar{g}}_{K}$, ${\bar{g}}_{Na}$, and ${\bar{g}}_{l}$ are the electrical conductances of the potassium, sodium, and leak channels, respectively. Additionally, ${\bar{g}}_{l}$ has a constant value for all neurons, while ${\bar{g}}_{K}$ and ${\bar{g}}_{Na}$ hold different values for different neurons. The reversal potentials of the three types of ion channel, namely $V_{K}$, $V_{Na}$, and $V_{l}$, specified the values for every neuron. The relevant constant parameters are summarized in Table 1.

The second term $I_{k}={\bar{g}}_{K}n^{4}\left(V_{m}-V_{K}\right)$ on the right-hand side of Formula (1) represents the current of potassium ions, while the third term $I_{Na}={\bar{g}}_{Na}m^{3}h\left(V_{m}-V_{Na}\right)$ refers to the current of sodium ions. The fourth term $I_{l}={\bar{g}}_{l}\left(V_{m}-V_{l}\right)$ corresponds to the leakage current. Accordingly, the current conduction in a single neuron is facilitated by various ion channels distributed throughout its membrane. In the neural network of *C. elegans*, parameters in the detailed expression of m,n, h vary for the different ion channels and are also contingent on their respective gene expressions. In the whole-brain model, certain crucial ion channels were selected, and their corresponding characteristics were explored in published experiments [32]. Furthermore, specific ion channels, which were previously thought to have non-negligible effects on membrane voltage alterations, were also included in this model. The following formula demonstrates an equation for the calcium channel, which has distinct parameters and slightly different forms compared with the previous channels in the HH model.

(2) $$I_{CA}={\bar{g}}_{CA}\cdot m_{CA}^{2}\cdot h_{CA}\cdot \left(V-V_{CA}\right)$$

where m and h have similar formulas to the other ion channels but different parameters of the expression of α and β.

It is of significance to note that some of the main potassium and calcium channels [22] exclusively involve specific genes associated with the stimuli sensing of mechanical forces, such as TRP-4 [33]. All the researched genes related to locomotion sensing are summarized in Table S1 in the Supplementary Materials. This study postulates a linear relationship between gene expression and the conductance values for each type of ion channel manipulated by distinct genes in all neurons, while the gene expression data for every neuron were extracted from the WormBase [34]. As a result, the conductance values for each neuron’s various ion channels can be determined by aggregating the necessary gene expressions and relevant data across all neurons. 

**Figure 2:**
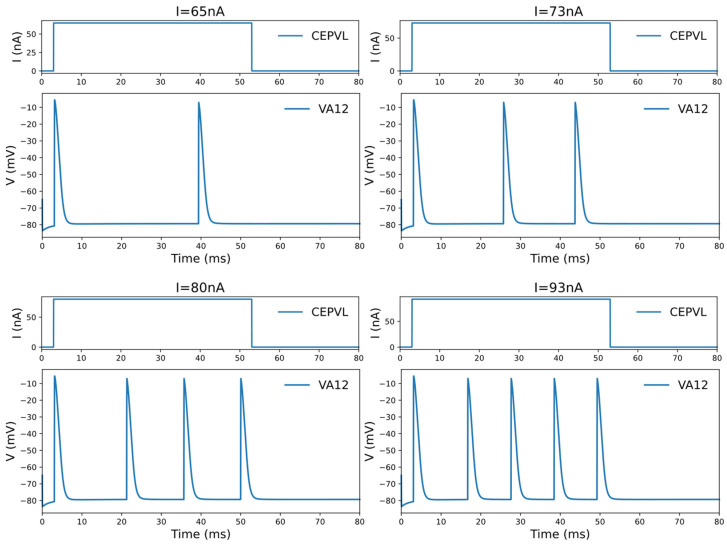
The oscillation phenomenon of the voltage response of the motor neuron under the current stimulation of the corresponding sensory neuron in one specific circuit, CEPVL-VA12. The frequency of the active spiking of the motor neurons’ voltage response grows larger with an increase in the current. The sensory neuron used for stimulation is CEPVL in the circuit, and the picture illustrates the changing forms of its external current stimuli. The motor neuron we observed is VA12, and the picture shows its responding voltage alterations, which show an oscillation phenomenon. The circuit is simulated in the whole neural network of *C. elegans*. The phenomenon containing the changes in spiking frequency can be used in the encoding of relevant continuous variables in a robot kinetic system. A more detailed picture is also shown in Video S1 in the Supplementary Materials.

The simulation of an individual neuron is based on a numerical computation of its action potential, which is generated by a current flowing across multiple ion channels distributed along the neurons, as per the HH model. In addition, the simulation determines the dynamic characteristics of the neurons’ potential membrane response. A significant finding in this context is the phenomenon observed in the HH model applied to *C. elegans*. Here, the voltage potential response displays an oscillation behavior when the leakage conductance falls below a critical value of 0.299406 [35], attributed to the HH model’s bifurcation. This characteristic manifests as follows: if the stimulating current applied to the neuron is low enough, it fails to elicit an action potential. Once the stimulation current reaches a critical level, the neuron will return to generating only one action potential. As the current surpasses a threshold, the neuron will fire multiple action potentials in a continuous and periodic pattern (see Figure 2). However, it will revert to generating a solitary action potential when the current increases beyond another critical point.

In addition to the model of single neurons, the neural network is formed by the synapse connections between neurons. These synapses are classified as either excitatory or inhibitory, depending on the impact that the presynaptic neurons have on the postsynaptic neurons. The mathematical formula that illustrates the synapse connections for this model is presented below.

(3) $$\{\begin{array}{c} I=g\times \left(v-e\right)\\ g^{\prime }=-\frac{g}{\tau }\\ g=g+w \end{array}$$

In this study, a simplified version of the synapse dynamic equation is utilized and compared to the original model [31]. The purpose of this simplification is to decrease the computational complexity while maintaining an analogous expression of the conductance parameter g. In terms of the expression, for the excitatory synapse, e=0, and for the inhibitory synapse, e=−80. A membrane voltage threshold of −30 mV was set for the chemical transmission of synapses, which means the postsynaptic neuron receives a current only when the voltage of the presynaptic neuron exceeds the threshold. We can construct a thorough computational model of the complete brain for simulation and analysis once we have the *C. elegans’* synapse connection data, which include the polarity and weights extracted from the previously presented website. We neglected gap junctions [36] in this model. This is because they have been observed to exist in almost every neuron of *C. elegans* [37]. As a result, running the simulation program with gap junctions makes it a more complicated process, while the research indicates that it has little impact on the membrane potential v [38]. Overall, neurons on three synaptic-linked levels contribute to the neural network. After the entire neural system is built, it is then used the program as a control element for the applied robot. The whole-brain model simulation codes are provided in the Supplementary Materials. Notably, the NEURON tool with Python was the simulation model tool used in this study [39].

#### 2.1.3. Control Circuit Identification

Underlying the complete network structure of *C. elegans* are specific neural circuits that regulate its movement and behaviors, which include forward and reverse locomotion, as well as direction-switching [16]. These circuits are primarily made up of sensory neurons that are functionally specialized in the control of certain motor neurons. To develop intelligent robot behaviors based on the mechanisms of *C. elegans*, it is essential to locate the controllable circuits in the neural network. For instance, previous studies indicate that ASH sensory neurons, as well as AVA, AVD interneurons, and DA motor neurons, can regulate reverse locomotion [16]. Additionally, the OLQ sensory neurons and RMD motor neurons can govern head withdrawal [40]. 

Through simulation, specific circuits can be identified by contrasting the voltage responses of all motor neurons. This differentiation manifests in the form of distinct voltage responses among specific motor neurons when subjected to current input alterations in their corresponding sensory neurons, while others exhibit no such dependency. By stimulating an individual sensory neuron with an input current with a large value range, the voltage responses across all motor neurons will be output through simulation for subsequent analyses and summaries. As a result, by continuously activating different sensory neurons, additional circuits are discovered within the whole-brain network model simulation, each with specific sensory and motor neurons. 

This study proceeded to investigate circuits’ dynamic properties by examining the voltage response features of relevant motor neurons through whole-brain model simulation. Some motor neurons exhibit voltage response oscillations, while others do not generate oscillations after activation. The simulation results are presented in Figure 2. This phenomenon can be used to determine the best approach to motor neuron decoding, such as continuous spiking frequency decoding or digital binary decoding, for decision-making. Additionally, the generation of the potential response oscillation behavior of motor neurons relies on the connected sensory neurons and the specific control circuit. For example, DA motor neurons generate voltage oscillations when activated by the sensory neuron ADEL, but only generate a single action potential when activated by the sensory neuron ASHL. Therefore, the dynamic responding features of motor neurons have to be derived from certain connected circuits in the controlling part of the whole-brain model.

All the useful circuits that were observed and researched, along with their corresponding sensory neurons and motor neurons, are shown in Table 2. Among them, all the sensory neurons are mechanosensory neurons. The range of voltage response oscillations caused by the current stimulus is summarized for use in the control section, meaning that the activation states of the motor neurons can be determined by the current stimulation value acting on the sensory neurons. The current stimulus is added to the sensory neurons within the corresponding circuit, and the value range contains a starting point to activate neurons in the circuit or the range required for the voltage response to generate oscillation behavior (shown in Figure 2). The obtained circuits were confirmed through prior research and experiments, indicating the presence of functionally controllable circuits in *C. elegans*. These findings will be utilized in the subsequent decoding and encoding methods presented in the control module.

Overall, all the functional circuits are summarized, along with their specific computing characteristics, including the relationship between the detailed voltage response of motor neurons and the value of the current input acting on sensory neurons. Therefore, the central functional control part that is discovered could be applied following more insights into the detailed dynamic response of several usable circuits, to achieve a practicable encoding and decoding protocol. The use of a correct and comprehensive whole-brain model guarantees the feasibility of its being utilized as the control core of intelligent robot behaviors, which also serves as the basis of this study.

### 2.2. Robotic Platforms

#### 2.2.1. Twelve-Legged Radial-Skeleton Robot

**Figure 3:**
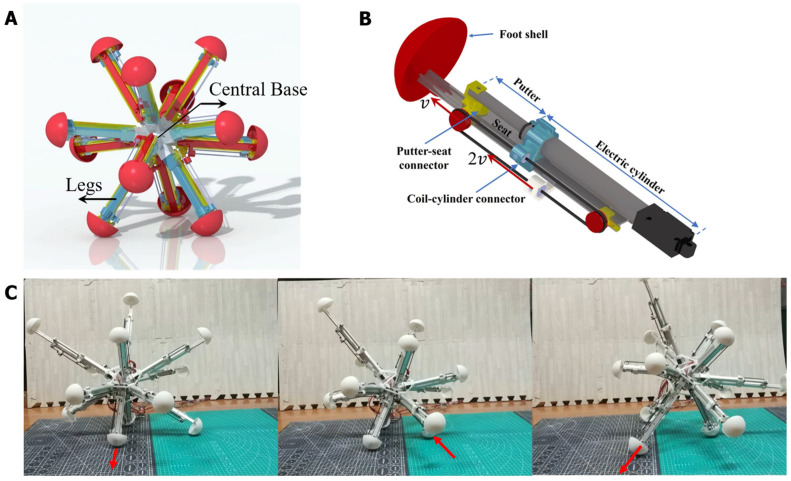
The physical structure of the radial-skeleton robot in this study. (**A**) The integral physical appearance of the robot. (**B**) The detailed configuration, including the stretching of one leg. (**C**) The movement of this multi-legged skeleton robot when stretching and shrinking its legs. The detailed instructions can also be read in the former paper [44].

A 12-legged radial skeleton robot is employed as the control object of the BNN model. This is a previously developed robot, which has a fully functional dynamical model [41,42,43]. The robot realizes the main functions of creeping, walking, obstacle avoidance, etc., which are as simple as the functions of *C. elegans*, so it was chosen as the applied object for the whole-brain BNN model. The radial-legged robot is composed of a robot base and legs fixed on one end of the base (as shown in Figure 3A) and its main application scenario involves complex terrain detection because of the unique features of its appearance. The robot base is designed to be a spherical shell, with 12 sleeves distributed uniformly on the surface to connect the legs. The amount of legs was selected as 12 through computation and testing, as this provided it with the best ability to walk on rough terrain [44]. More importantly, the robot legs can stretch out and draw back, relying on the two-way pulley mechanism shown in Figure 3B.

As shown in Figure 3B, taking the first section as the study object, the pulley fixed on the first section extends to the second section at a speed of V. A point on one side of the coil is fixed on the robot base and moves to the right at a relative speed of V, the same as the first section, while the coil on the other side moves to the left at the same speed as the first section. Adding the convected velocity of the first section, the second section finally elongates at the speed of 2 V. In total, the robot reaches a radial expansion ratio of 2.08, which provides it with a better rolling ability [44]. The foot shell is installed on the bottom end of the slide rail, which has a hemispherical design so that the foot can make contact with the ground omnidirectionally. It is relatively rough so that the sliding of the foot can be decreased in real experiments. In total, the movement of this radial skeleton robot means that that the center of gravity moves with the support triangle, composed of three grounding legs with an expanding length. Then, the robot will roll in one direction (as shown in Figure 3C).

#### 2.2.2. Kinetic Model of the Robot for Simulation

We built a kinetic model corresponding to the robot’s motion pattern [17]. Since the main mass of the robot is concentrated in the central base and the motors and their electric cylinders are firmly connected to the base, the change in the mass distribution of the robot is not significant during movement. As a result, in this paper, the robot is simplified as a solid ball with uniformly distributed mass when calculating its moment of inertia. We took the robot as a multi-rigid-body system in this dynamic model, and we considered the changing length of its legs as the time-varying constraints. Consequently, the state variables of the central rigid body can describe the whole robot movement. They are as follows:

(4) $$q={\left[x,\;y,\;z,\;\lambda_{0},\;\lambda_{1},\;\lambda_{2},\;\lambda_{3},\;v_{x},\;v_{y},\;v_{z},\;\omega_{x},\;\omega_{y},\;\omega_{z}\right]}^{T}$$

In the formula, x, y, and z denote the coordinates of the center of mass of the robot under the Cartesian coordinate system, which is also an inertial reference system. $\lambda_{0},\;\lambda_{1},\;\lambda_{2},\;\lambda_{3}$ represent the robot attitude as quaternion. $v_{x},\;v_{y},\;v_{z}$ represent the velocity of the center of mass under the inertial reference system. $\omega_{x},\;\omega_{y},\;\omega_{z}$ represent the angular velocity of the robot under an accessory coordinate system. 

Neglecting the mass of stretching legs, the acceleration of the center of mass in the simulation environment can be determined by the following motion theorem:

(5) $${\left[\dot{x},\dot{y},\dot{z},{\dot{v}}_{x},{\dot{v}}_{y},{\dot{v}}_{z}\right]}^{T}={\left[v_{x},v_{y},v_{z},\frac{F_{x}}{m},\frac{F_{y}}{m},\frac{F_{z}}{m}\right]}^{T}$$

where m means the mass of the robot; $F_{x},F_{y},F_{z}$ are the external forces of the robot, including gravity and contact forces. 

Usually, we use Euler angles to describe the attitude of an object. However, the spherical multi-legged robot can change its motion attitude over a large range during rolling movement, which means it is easy for the robot to be in a singular configuration. As a result, in the dynamic model, we adopted a quaternion algorithm to represent the robot attitude. The derivative of the quaternion is based on the following formula:

(6) $${\left[{\dot{\lambda }}_{0},{\dot{\lambda }}_{1},{\dot{\lambda }}_{2},{\dot{\lambda }}_{3}\right]}^{T}=\frac{1}{2}\left[\begin{array}{c} \begin{array}{ccc} -\lambda_{1} & -\lambda_{2} & -\lambda_{3}\\ \lambda_{0} & -\lambda_{3} & \lambda_{2}\\ \lambda_{3} & \lambda_{0} & -\lambda_{1} \end{array}\\ -\begin{array}{ccc} \lambda_{2} & \lambda_{1} & \lambda_{0} \end{array} \end{array}\right]\left[\begin{array}{c} \omega_{x}\\ \omega_{y}\\ \omega_{z} \end{array}\right]$$

The angular accelerations of the accessory coordinate system of the robot can be calculated using the Newton–Euler Dynamic Equation.

(7) $$\{\begin{array}{c} J_{x}{\dot{\omega }}_{x}+\left(J_{z}-J_{y}\right)\omega_{y}\omega_{z}=M_{x}\\ J_{y}{\dot{\omega }}_{y}+\left(J_{x}-J_{z}\right)\omega_{x}\omega_{z}=M_{y}\\ J_{z}{\dot{\omega }}_{z}+\left(J_{y}-J_{x}\right)\omega_{y}\omega_{x}=M_{z} \end{array}.$$

where $J_{x},J_{y},J_{z}$ are the moments of inertia of the robot and $M_{x},M_{y},M_{z}$ are the momentum acting on the robot, along with their projection components on the inertial principal body’s axis coordinate system. To simplify the calculations, take the robot as a sphere with a uniformly distributed mass. Therefore, the moment of inertia of the random axis that passes through the center of mass is equal. Then, the formula will be as follows:

(8) $$\left[{\dot{\omega }}_{x},{\dot{\omega }}_{y},{\dot{\omega }}_{z}\right]=\left[\frac{M_{x}}{J_{x}},\frac{M_{y}}{J_{y}},\frac{M_{z}}{J_{z}}\right]$$

After determining the robot’s kinematics, its entire kinetic simulation is created through computer programming. This illustrates the robot’s motion for a few simple movements in the initial state. Additionally, interfaces are established between the robot simulation and the biological neural network model, which involve encoding and decoding codes, and real-time SOCKET communication [45] between two programming systems. Following the simulation model’s construction, an experimental platform will be built for the robot to conduct physical tests of practical movement and control.

#### 2.2.3. Construction of the Experimental Platform

**Figure 4:**
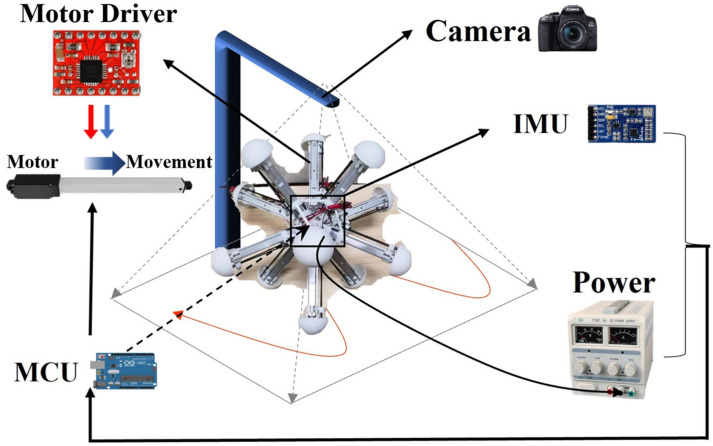
Total hardware control process and system of the robot used in the experiment.

The robot was manufactured and assembled according to its mechanical design, and equipped with the essential sensors and necessary hardware based on the experimental preparation. The necessary motors were also installed to control the legs, which were fixed at one end to the central base, as shown in Figure 3B. To achieve closed-loop control, the robot control system was divided into three parts: perception, decision, and motor execution. The Intertial Measurement Unit (IMU) measures the angle, angular velocity, acceleration, and robot attitude in real-time and transmits the data to the microcontroller via serial port communication. The Arduino microcontroller serves as the Microcontroller Unit and is responsible for the calculation function; it converts the sensor’s original input information into the speed and direction of each leg movement. These two parts determine the robot’s competence in proprioception and basic control, preparing for the latter experiments with the BNN control. The robot uses a power supply to power the Microcontroller Unit (MCU), the IMU, the motor controller, and the motors. The power supply and all the devices are installed in the central base, along with the necessary counterweight to ensure that the center of gravity is approximately the physical center of the base. In addition, the research used imperative hardware device modules to conduct field experiments in terms of the whole platform, such as a camera. Figure 4 shows all the hardware and its connection to the experimental platform containing the necessary sensors. The power, IMU and MCU were all installed in the robot’s center and connected with each other, with the motor drivers and motors distributed across the 12 legs so that the experiment could operate correctly. The camera was placed above to record the experiments. Moreover, to ensure a high degree of fidelity between the simulation results and the actual motion of the robot, the parameters of the robot in the simulation environment were the same as those of the real robot (Table S2 in the Supplementary Materials). The BNN model can communicate with the serial communication module of the robot’s central panel remotely in real-time. As a result, simulations and experiments could both be carried out in this research. 

Based on the establishment of the experimental platform, essential equipment was installed, and preliminary tests of the robot were conducted to assess its basic locomotive capabilities. The simulation model of the whole-brain neural network of *C. elegans* and the robot kinetics was tested before the further process of control, determining its communication modules and operation efficiency in terms of real-time simulation and experiments. As a result, the integrated models and platforms collectively affirmed the viability of the subsequent control methodology.

## 3. Results

### 3.1. Visualization of the Whole-Brain BNN Model

**Figure 5:**
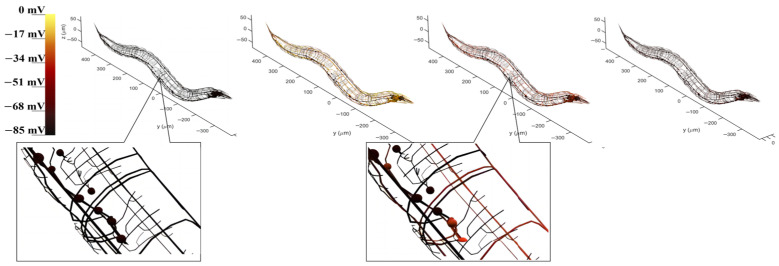
The visualization of the voltage responses of the whole neural network model to the stimuli acting on the sensory neuron CEPVL. The color change corresponds to the neuron voltage changing from −85 mV to 0 mV, where yellow means the voltage is close to 0 mV and black means the voltage is close to −70~−85 mV. Therefore, yellow represents the action potential and black represents the resting potential. The four pictures showing the four stages of the voltage change at one moment were taken continuously. In this process, the neurons go from resting potentials to action potentials and then back to resting potentials, which corresponds to the circuit changing from black to yellow and then gradually turning back from yellow to red and black. A more detailed diagram is also shown in Figure S2 in the Supplementary Materials.

The BNN model served as the central control of the robot. We first present the utilized BNN model in detail, along with the control circuit’s highlights. The visualization of the BNN allows for the model simulation results for the previously determined circuit within the whole-brain model, which contains neuron morphology and voltage response projection, to be objectively examined. The whole-brain neural network of *C. elegans* is plotted, incorporating the morphology data of each neuron to ensure an accurate representation. During model simulation, the potential electrical membrane response of neurons can be recorded when stimulated by current inputs from sensory neurons. By tracking the state of these recorded neurons, a corresponding color change can demonstrate the dynamic electricity transduction between neurons. This reveals that many neurons are activated through the current stimulation of sensory neurons during the entire process. Figure 5 illustrates the control circuit with CEPVL, showcasing the electricity transduction visualization as well as the morphological data of each neuron, including sensory neurons, interneurons, and motor neurons. The voltage response of neurons corresponds directly to color alterations, so the circuit can be highlighted in the whole-brain atlas by showing the color changes at different stages when activated by a stimulus. This means that the voltage responses of all the neurons within the circuit are connected to their color (black means static and yellow means peak in voltage). The activating processes of neurons dictates that voltage changes from a static state to an activated state suddenly and returns to a static state relatively slowly, leading to a rapid color change from black to yellow and back to red and black based on neuron visualization. Consequently, the plot results dhow the circuit’s dynamic characteristics, along with the neural network model’s overall morphology and configuration. All the morphological data of the whole-brain neural network neurons were extracted from the NeuroMorpho.Org [46] and then plotted using MATLAB (https://www.mathworks.com/products/matlab.html).

### 3.2. The BNN Model Controls Robot Orientation

#### 3.2.1. Mechanism by Which BNN Controls the Robot

**Figure 6:**
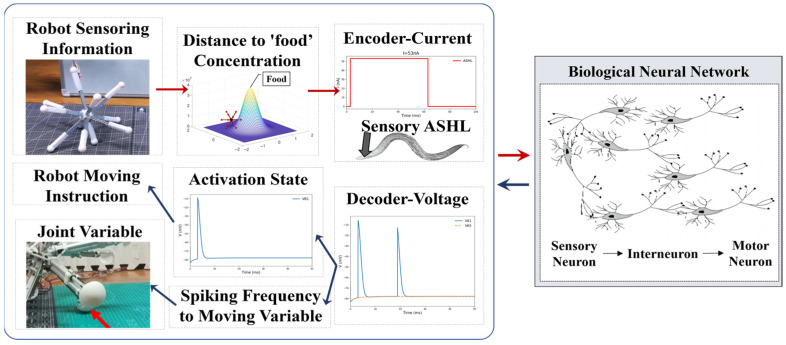
The encoding and decoding method used to connect the robot system and the biological neural network system. The encoding current is derived from the robot’s sensing information, such as its distance from the ‘food’ target point, which will be elaborated in the following section, which discusses how to transform the distance into the input current. Once the biological neural network receives the input, it starts to complete the whole-brain network simulation. The simulation results of the motor neurons will be decoded into movement variables or a movement command for the robot to complete the next motion step.

When employing the BNN model for robot control, it is imperative to derive the basic control mechanism from the biological intelligent control system. In general, the mechanism by which the biological neural network controls *C. elegans*’ behavior is relatively clear [47]: initially, sensory neurons detect environmental stimuli, such as changes in food concentration, and generate action potentials. From there, through signal transduction, the sensory neurons activate their postsynaptic neurons, including interneurons and motor neurons, in particular control circuits. Muscles will generate either a shrinking or relaxing movement to facilitate the responsive motion of *C. elegans*, driven by the electrical transduction from motor neurons. This transduction, which operates based on three layers of neurons and muscles, determines the BNN’s capacity to control the creature’s intelligent behaviors. Therefore, with reference to the BNN model mechanism, the robot’s control method can adopt this entire information processing approach, using the biological neural network’s behaviors and dynamic features. This approach includes transforming the signals of biological environment stimuli into physical sensing signals for the robot and transforming the micro-behavior of biological muscles into physical information for robot motion. By replacing the genuine biological stimuli experienced by sensory neurons, the robot sensors can encode the relevant input into a stimulation current format based on the BNN model. Furthermore, the method employs the diverse dynamic features of motor neuron voltage responses to decode them for the control of robot joint motions, substituting actual biological muscles. This is because the lack of clarity in the mathematical relationship between motor neurons and muscles [48] limits the ability to directly decode from the muscles. In this procedure, the data from the robot sensors will be transformed into current stimulation and used as the input stimulus for the sensory neurons. The robot joints, controlled by the motor neuron simulation output, will act like the muscles of *C. elegans*. Figure 6 illustrates the conceptual framework of this comprehensive research method.

In terms of the form of the current stimuli, previous research suggests that certain mechanosensory neurons respond to particular current stimuli patterns [22]. However, based on the simulation results derived from the network model, it is not appropriate to use this method in the current model. Instead, a constant value for the current was input over a continuous time (as shown in Figure 6). It was determined that the voltage response undergoes continuous changes following simulation, providing insight into its dynamic properties. Therefore, according to the specific dynamic characteristics of motor neurons, the voltage response can be directly decoded into leg length changes or motion instructions for a multi-legged robot. In the latter methods, the motion instructions can be transformed into the command of electrical motors in an experiment or the command of each leg’s extension length value based on the inverse kinetics during the simulation. The binary activation state of non-oscillatory motor neurons after activation can be transformed into discrete variables for movement instructions. The former approach decodes the spiking frequency of motor neurons’ voltage responses into continuous variables, including the joint movement variables that correspond to the muscle’s relevant motion variables in *C. elegans*, based on their oscillation behaviors. The encoding and decoding processes are detailed in Figure 6.

In addition to implementing the fundamental combination approach to the BNN model and the robot system, our attention was also directed toward selecting appropriate sensory and motor neurons for the control loop in this study. For *C. elegans*, a range of composite control circuits, incorporating operational sensory and motor neurons, facilitate its intelligent behavior control, such as foraging and obstacle avoidance. Consequently, except for the basic motion control modulated using the motor neurons, the specific targeted control circuits should be determined, along with their kinetic characteristics, so they can be combined with robot intelligent control, as well as the sensory neurons and motor neurons that are to be encoded and decoded. For instance, the sensory neurons ADEL/R are dopaminergic nose touch mechanoceptors. They modulate the locomotion behavior in response to the presence of food through textural mechanosensation [49]. When a current stimulus reaches an ADER sensory neuron ranging from 59 nA to 68 nA, the oscillations in voltage response will take place in the motor neurons containing DA, DB, VA, VB, and VD (as shown in Table S2 in the Supplementary Materials). By setting the value of leakage conductance, the spiking frequency of voltage response oscillations in motor neurons can be continuously decoded according to the changes in the length of robot legs (similar to what is shown in Figure 2). By choosing 12 neurons, the changes in leg length can be connected to the voltage decoding of motor neurons. Once all the leg lengths are determined, the robot can move in a certain way. Consequently, it is feasible to use the BNN model to control the basic motion of the robot by determining the appropriate circuit (the realization of a platform focusing on the whole mechanism, including the interaction between the two simulation systems, is also shown in Figure S1 in the Supplementary Materials). Furthermore, a particular intelligence-based conduct is predominantly achieved in the robot through the development of field missions that pertain to brain-inspired intelligence, stemming from the basic mobility feature.

#### 3.2.2. Innate ‘Foraging’ Behavior Control

Inspired by *C. elegans*, robot control could be achieved through an imitation of biological intelligence to generate similar intelligent behaviors in the robot. For instance, *C. elegans* can squirm to food slowly by detecting and following an increasing gradient of food concentration [50]. Combining this with basic robot functions, such as locomotion and rolling on the ground, could enable similar mission planning for the robot, allowing it to move toward a target point. In the natural habitat of *C. elegans*, alterations in food concentration rely solely on the distance between the worm and a food source [50]. Consequently, for practical control purposes, we can also convert the distance between the robot and its target point into a ‘food’ concentration to link the BNN control mechanism with the robot.

For *C. elegans*, when sensory neurons detect a change in food concentration, the whole neural network will transmit the corresponding signals directly to the muscles distributed along its body, causing it to perform undulatory or turning motions in response to the altered food environment [51]. For instance, when an increasing concentration of food is nearby, the whole neural network and muscle activities are triggered to move *C. elegans* closer to the food. 

In alignment with the neural behavior, the robot uses an indicative input signal to ascertain its proximity to the target point. This mimics the intelligent mechanisms of *C. elegans*, detecting the robot’s movement direction in relation to the increasing ‘food’ concentration. Therefore, considering the ‘food’ concentration changes, the input current will directly stimulate the sensory neurons. This, in turn, allows the BNN model to control the robot by altering the distance to the target point. Meanwhile, the robot’s motion can be controlled by decoding the joint moving variable values from the voltage response of specific motor neurons in the chosen circuit, or by following moving instructions generated by the BNN to produce intelligent motion. An appropriate decoding method is essential in this control mechanism, and we conducted preliminary experiments to test two decoding methods.

Building on the previous idea, we began by using the circuit containing the oscillation phenomenon for motor neurons, which allows for the continuous decoding of leg extension length. This is necessary because the output of the BNN needs to be decoded according to the changes in the leg length of the robot in the former idea. We selected 12 motor neurons in a chosen circuit ADER-VA, which satisfies the requirements for this method, and connected them with the 12 robot legs. To align this with the input current range of oscillations in sensory neurons such as ADER, it is necessary to limit the current stimulation range to a small interval. Additionally, the input current value is correlated with the distance between the robot and the target point. Therefore, during the robot’s movement, the control process should follow the following steps: if the robot is further from the target in a previous step, the current stimulated on the sensory neuron will be relatively smaller, in alignment with the encoding. Regarding the oscillation phenomenon, a low current will result in a longer period of action spikes. The decoded output should assist the robot in moving closer to the target in the next step. If the robot moved closer to the target in a previous step, the current will increase, resulting in a higher frequency of action spikes. This change should also aid the robot in moving closer to the target in the next step. 

In this procedure, to determine the mathematical correlation between the voltage response of the motor neurons and the leg extension length, a differential equation expression [10] is used. However, the initial findings have shown that the robot cannot perform well using this muscle-joint motion method [10], which directly connects the response of the 12 motor neurons with the 12 legs’ extension motions. This indicates that the robot exhibits smooth movements during the first few seconds, but it fails to display intelligent movement thereafter. Ineffectual moving results were obtained, which were controlled by directly encoding the spiking frequencies to the joint variables of the robot. (A detailed video of the robot moving in the simulation is also shown in Video S2 in the Supplementary Materials, with a failed moving situation being shown at the end.) This is because the robot has a distinct kinematic model to that of *C. elegans*, making it impractical to apply the whole-brain model to the robot’s self-intelligence control without considering the robot’s specific moving properties. Therefore, intelligent ‘foraging’ control of the robot cannot be achieved solely by employing the direct decoding method and the ADER circuit.

For the latter control mechanism, since the robot has a distinctive movement system to that of *C. elegans*, it is practicable for the biological neural network to make the policy decisions, aiming to achieve a specific intelligent function of robot motion, rather than directly projecting the BNN onto the robot’s joint movement variables. For this method, the policy can be the direction in which the robot should move to achieve the ‘foraging’ behavior. When employing the BNN as the direction control element for the robot system, integrating the robot’s kinetic model is compulsory for an all-inclusive closed loop. This ensures the robot’s motion is controlled. As per inverse kinetics, once the direction of the robot motion is established, variables for each joint can be resolved so that the robot can move in the predetermined direction to complete the whole ‘foraging’ behavior. It should be noted that the decision of a specific direction can be an absolute direction, indicating the placement of the ‘food’, or a relative direction that is based upon exploring the environment. 

Robots can construct an absolute coordinate system by employing computer sensors. However, it is hard for the biological neural network model of *C. elegans* to decide on a precise direction in a three-dimensional space. This study has demonstrated that neural circuits like CEPVL-VA, which can be decoded to a continuous direction angle variable, are incapable of performing the task of moving a robot to an arbitrary point using methods based on absolute geometry. However, in the foraging behavior of *C. elegans*, the worm decides its locomotion direction step by step according to the feedback regarding food concentration [50], even though it is not equipped with an advanced navigation system. In this process, it will follow its previous direction or move forward if the food concentration is increasing. However, it will have a reversed motion when the food concentration determined in the previous step is decreasing [21]. Consequently, it can move in the direction of the increasing food gradient without completely determining its environment, although it may not move along the shortest path.

**Figure 7:**
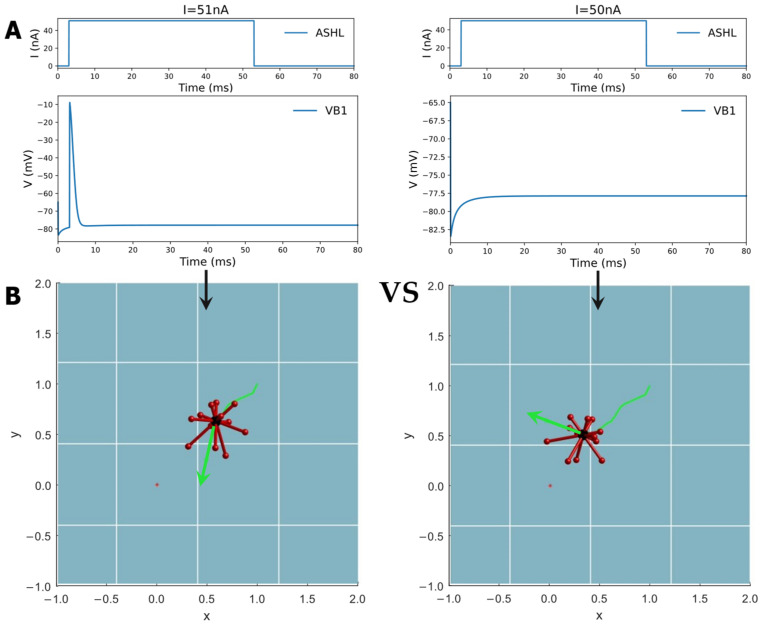
The circuit ASHL-VB1 searches for the highest concentration point in the direction of the gradient increase. The circuit possesses dynamic characteristics where the motor neuron (output neuron) is activated by the current stimulation of the sensory neuron with no oscillations, and the control results are shown for the simulations of a moving robot. If the current stimulating the sensory neuron is higher than the threshold, the motor neuron will be activated. If the current is lower than the threshold, the motor neuron will generate no acting potential. The threshold of the current input corresponds to the moving state of the robot. Through simulation, the value of the acting current was found to be 51 nA. The activation state of motor neuron VB1 can also be identified through Python programming. From (**A**) to (**B**), the Figure shows the detailed control mechanism of this method. According to the movement direction signified by a green arrow in the robot simulation picture, if motor neuron VB1 is activated, the direction in the next step is the same as that in the previous step. However, if motor neuron VB1 is not activated, which means the robot is further away from the target point, then the robot will be given a new direction until it finds a direction that allows it to move closer to the target. A video of the robot’s movement is shown in Video S3 in the Supplementary Materials.

By emulating this behavior, the study designed a digital environment to simulate a robot moving toward a ‘food’ concentration. The value gradient transformation was modeled on a Gaussian distribution, with a lower food concentration value being indicated when the robot is further away from the target point. Consequently, the study based on this imitation of ‘foraging’ behavior concluded that if the ‘food’ concentration is higher in the current step than in the previous one, the robot will continue to move in the same direction. When the ‘food’ concentration drops below that of the previous step, the robot will select a random direction as its next step until it detects an increase in concentration. In this process, the biological neural network carries out the function of decision-making. Thus, a designated circuit, ASHL-VB1, which is decoded into a binary digital signal of either 0 or 1, is chosen (as is evident in Table 2). The electrical dynamic aspects of this circuit indicate the absence of any oscillation phenomena with only one action potential or none, as shown in Figure 7A. ASHL represents the primary nociceptor, eliciting avoidance responses to noxious stimuli. The alterations in concentration values are decoded directly into the current that stimulates the sensory neuron ASHL. If the concentration rises, the current surpasses the threshold value needed to activate the corresponding motor neurons. If the concentration declines, the current falls below this value. The mathematical expression of changes in concentration is as follows:

(9) $$f\left(x\right)=10000000*e^{-2*x^{2}}$$

where *x* represents the distance from the target point to the robot and f(x) means the ‘food’ concentration at that point. The reason why the expression has a very large coefficient is that when the displacement of the robot between two steps is extremely small, the current input for the sensory neuron will still pass the threshold if the robot is moving closer to the target point. Meanwhile, f(x) is decoded directly to the stimulation current acting on the sensory neuron.

(10) $$I\left(x_{i}\right)=51+f\left(x_{i}\right)-f\left(x_{i-1}\right)$$

where $x_{i}$ means the current state, $x_{i-1}$ means the previous state, and the value 51 is determined according to the current threshold.

**Figure 8:**
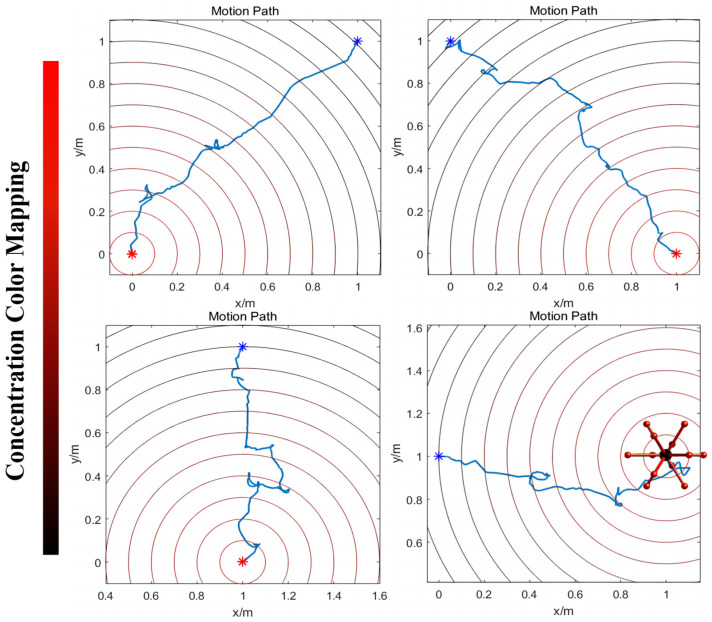
The simulation movement results of the robot moving to a target point with the highest ‘food’ concentration. Overall, the robot can move from the initial point to the target with some spiraling states. The blue point means the initial point and the red point means the target; the robot’s movement path was plotted between these two points. On the left side of the figure is the ‘food’ concentration color map. Red means a higher concentration and black means a lower concentration. The experiment uses the unit of one meter. The results demonstrate that the robot can move autonomously from the lower ‘food’ concentration point to the ‘food’ under the control of the BNN model of *C. elegans*. Additionally, the movement details are shown in Video S3 in the Supplementary Materials.

When the robot approaches the target point, the concentration of ‘food’ increases, leading to a rise in the current that exceeds the required threshold for it to activate motor neuron VB1. This activation is decoded into a movement instruction, prompting the robot to follow the direction of the previous step. Conversely, as the robot moves away from the target point, the concentration of ‘food’ decreases, resulting in motor neuron VB1 remaining inactive. The resting state is interpreted as the motion command to arbitrarily choose a new direction until the robot begins to move closer to the target (the detailed process is shown in Figure 7). Figure 8 displays the simulation results when controlling a robot to move toward various target points from different initial points, moving in four designated directions. The robot may exhibit some spiraling movements, but it effectively reaches the target point by following the direction of the increasing gradient. In summary, this method demonstrates that robots can generate autonomous, intelligent orientation behaviors if the biological neural circuit is used to control their policy-making components, similarly to the foraging of *C. elegans*. This provides an intelligent control method that improves robots’ biomimetic intelligent sensing and decision-making abilities, including their ability to navigate to target points in unfamiliar environments without the need for complex sensors. The BNN model presents an autonomous and pragmatic approach to directing a robot’s intelligent behaviors in a designated mission.

#### 3.2.3. Omnidirectional Locomotion Control

*C. elegans* can not only decide on a movement direction but can also manipulate the muscle motion to make its whole body move in the given direction. Therefore, the second method by which the biological neural network can achieve intelligent movement is to direct the self-organized movement of the robot once the movement instruction has been determined. If the direction of the robot movement is determined, the joint motion is controlled using the BNN model, inspired by the control of *C. elegans*’ muscles via motor neurons to generate movement. Thus, it is also possible and justifiable to utilize the motor neuron output of the biological neural network to control the joint motion, which, in this study, refers to the extension of the robot’s legs when the movement direction is predetermined and certain, thereby replacing the inverse kinematics.

**Figure 9:**
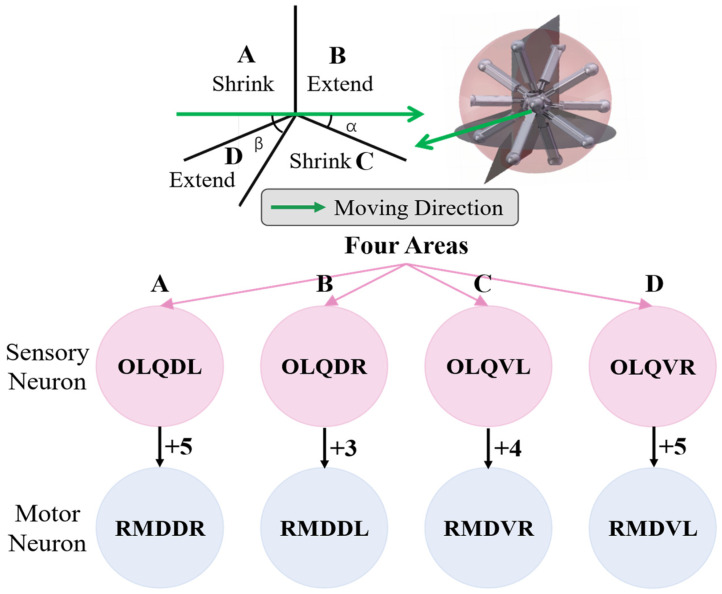
The design of four specific circuits to control the legged robot’s motion in a particular direction. The robot is divided into four parts using four planes vertical to the moving plane. As is shown in the picture, the detailed motion of the legs in these four parts can be derived from inverse kinetics so that the four districts exactly correspond to the four control BNN circuits. The symbol and number between the sensory neurons and motor neurons refer to the polarity and weight of their synapse connections, respectively.

The model for the locomotion of the robot in a specific direction involves dividing the robot into four regions perpendicular to the heading direction plane, as depicted in Figure 9. In Region A, the legs should shorten, while in Region B, the legs should elongate. In Region C, the legs should also shorten, and in Region D, the legs should elongate. This process will facilitate the gradual movement of the robot, making it roll in the given direction. For the purposes of BNN control, the biological neural network was integrated into the control system by establishing a mapping relationship between the four leg regions and the corresponding instructions for the adjustment of leg length. The four circuits of the OLQ sensory neurons and RMD motor neurons were selected to achieve this objective. The OLQ sensory neurons regulate the head-withdrawal reflex, with the RMD motor neurons serving as the synaptic targets. It is noticeable that the chart shows that a particular OLQ neuron regulates an individual RMD motor neuron without oscillation behaviors, as per the simulation outcomes. Specifically, OLQDL controls RMDDR only, OLQDR controls RMDDL only, OLQVL controls RMDVR only, and OLQVR controls RMDVL only. The robot simulation environment enables the derivation of the leg’s corresponding region. Then, the current stimuli of the four OLQ neurons encode the four regions, and the output of the four RMD motor neurons can be decoded into the length change in the legs in the corresponding regions. To activate these sensory neurons, the stimulation current is increased beyond the stimulation threshold, as shown in Chart 1. The legs in regions B and D stretch only if RMDDL and RMDDR are activated. The legs in regions A and C should shorten if RMDVL and RMDVR are activated. Then, the robot can move incrementally in a particular direction using this control method. The study tested linear movements in directions of 0, 45, 90, and 135 degrees. The results indicate that the robot moved effectively in the simulation. It can be concluded that the robot successfully achieved a generalized self-controlled motion in a specific direction in simulation environments.

### 3.3. Experimental Validation of the BNN Control

**Figure 10:**
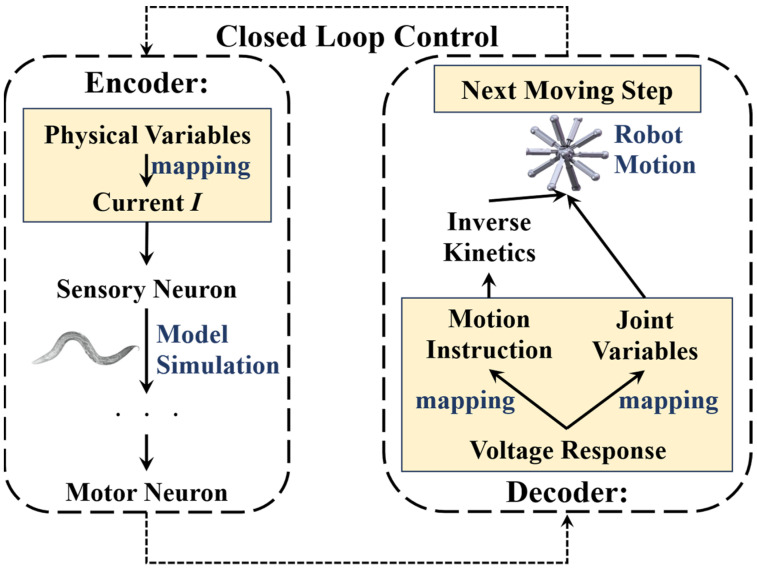
The combination of operation mechanisms for the BNN control system and the robot movement system. The physical variables of the input encoding of the BNN model could be the ‘food’ concentration changing or the districts of the robot legs. The inputs are mapped with the current stimulation on sensory neurons. After we obtain the voltage response of motor neurons in a model simulation of the BNN model, it will be decoded into the motion instructions or joint variables of the robot according to the designed missions. Then, the robot can complete the next step’s motion and transmit the necessary information to the BNN model for the next step’s simulation and control. This whole process is closed-loop and continuous in real-time.

The two control mechanisms were obtained from the simulation results of the methods containing an applicable encoder and decoder for the BNN system and the robot system. It was concluded through the simulation that the BNN model can not only help the robot orientate in particular directions but also helps it to control its self-locomotion. The two different mechanisms are specifically designed to achieve different goals and control targets in terms of the robot’s step control. As a result, the two mechanisms can be combined sequentially to achieve total closed-loop control for the intelligent ‘foraging’ behavior of the robot (the closed-loop control description is shown in Figure 10). To test and verify the feasibility and performance when using the biological neural network to control the robot in practice, experiments aiming to achieve movement results were carried out, which focused on self-locomotion control. 

**Figure 11:**
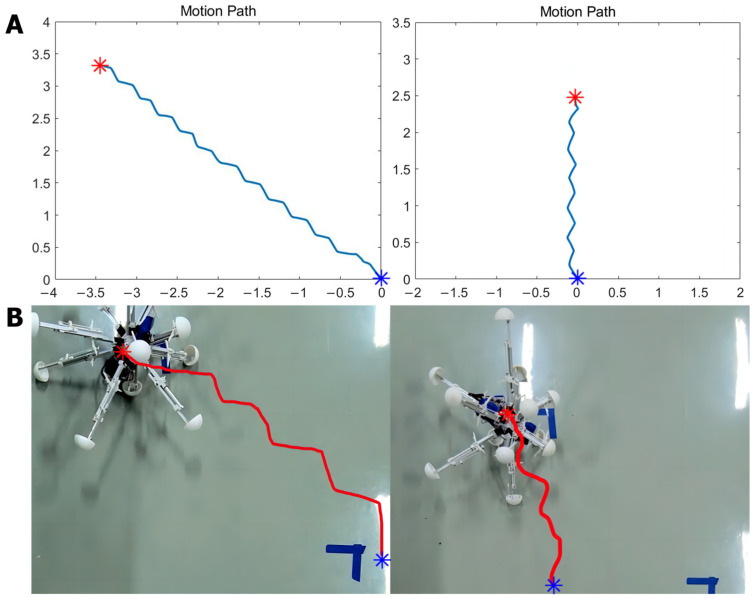
The simulation and experimental results of the robot movement controlled by the four-region control mechanism shown in Figure 9. The control mission was set to make the robot move along a straight line. The line has two directions: 135 degrees and 90 degrees to the x axis. In the illustrated figure, the robot can be seen to move precisely along the straight direction, step by step, during the experiments, according to the recorded movements. In (**A**), the blue lines show the movement track of the robot in the simulation. The experimental results show only slightly different effects compared with the simulation. Blue points represent the starting points, and red points represent the destination points. The red lines are the plotted movements of the robot in the experiments. The detailed robot movement experiment of (**B**) is also shown in Video S4 in the Supplementary Materials.

Firstly, in the experiments, in order to facilitate real-time data exchanges between the BNN model platform and the Microcontroller Unit (MCU) embedded within the robotic system for the purpose of transmitting locomotive instructions, the remote serial communication module in the central control panel of the robot is employed. This entails the necessary programming within the MCU, encompassing the incorporation of communication protocols and codes essential for seamless data transmission. Specifically, the experiment aims to validate the self-locomotion robot control when moving in a straight line. The length signal changes for every leg that are received by the BNN simulation model are directly output to MCU in the panel to control the electric motor in every leg. Then, the motor driver installed on the legs will control the leg to complete the corresponding movement, such as stretching or shrinking. Simultaneously, the leg region’s information is also remotely transmitted to the BNN simulation model to be encoded as the input for the sensory neurons. As a result, for every movement, the robot first transmits the leg region information received by the IMU module to the BNN programming; then, the four-region control circuits are run with the sensory neurons stimulated by the current input. The outputs of the four motor neurons (RMDDR, RMDDL, RMDVR, and RMDVL) are projected to the movement length of the legs in the four regions, and then the information about leg length changes is transmitted to the MCU of the robot system. The relevant module passes the instructions to the motor drivers to control the leg movement. Finally, the robot motion is completed using this step-by-step, closed-loop control mechanism. This entire process means that the real-time BNN model could operate in parallel with the robot movement system, ensuring that the BNN model of *C. elegans* can control the robot remotely. After setting certain locomotion directions for the robot in the MCU in advance, of 135 degrees and 90 degrees, the experimental results show that the robot can successfully move in the required direction. The entire movement was recorded, and a video-based motion path was plotted. In Figure 11, the robot’s movement is plotted, explicitly showing the robot’s direction, at 135 degrees or 90 degrees to the x axis, under the control of the *C. elegans*’ BNN model. When compared with the simulation environment, where the robot demonstrated high accuracy in moving along the line, the physical robot could move in a basic directional manner during real experiments, as evidenced by the plotted traces. Although the movements were not as smooth and precise as the expected target, the experiment verifies the validity and functionality of using a biological neural network to ensure the intelligent, self-controlled behavior of a robot moving in a straight line. 

**Figure 12:**
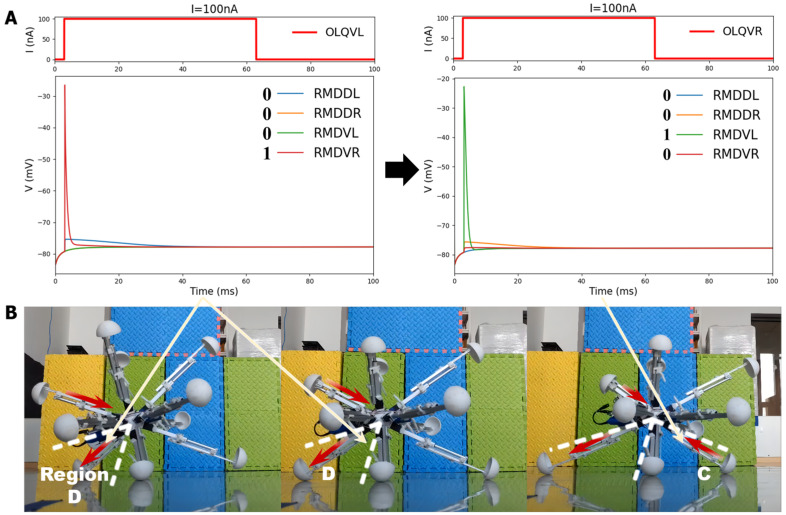
The detailed stretching motion of the robot legs in three continuous steps when using the BNN control of *C. elegans* to move in a straight line. In (**A**), the two diagrams illustrate the responses of the corresponding four motor neurons when stimulated by the sensory neurons OLQVL and OLQVR, connected to regions D and C in the robot. The activation state of the four motor neurons is also labeled in the diagram with 0 or 1 (1 means that the motor neuron is activated by the circuit and 0 means the motor neuron is in a resting state). The activated motor neuron is also connected to the changing instructions transmitted to the robot legs: 1 means that legs in the corresponding B&D region (RMDDR and RMDVR are decoded into these two regions) will extend, while the legs in the A and C regions (RMDDL and RMDVL are decoded into these two regions) will shrink. In this way, the robot can move in a particular direction. In (**B**), the red arrows denote the length-changing direction of the legs, shown by the white lines. Firstly, the legs in region D are controlled to continuously extend, and then the legs in region C begin to shrink, causing the robot to move in the correct direction. The results demonstrate that the robot follows the four-region rule, and the robot control is based on the four specific BNN circuits shown in the figure. In detailed procedure, leg movement in regions C and D is specifically controlled by the OLQVL-RMDVR circuit and OLQVR-RMDVL circuit.

The experiment also recorded the specific motion of the legs in the four districts to illustrate their detailed moving state when the robot moved in a particular direction, as shown in Figure 12B. This reveals that the robot’s leg movements strictly adhered to the basic four-region rule controlled by the circuits OLQ-RMD, which contained four control circuits in the BNN model, enabling the entire robot to move in the desired direction. The detailed process of each leg’s moving state, as determined by the four corresponding motor neurons’ voltage response features in the circuit, is also shown in Figure 12A,B. For instance, the leg movement in Region D was controlled by the OLQVL-RMDVR circuit: when adding an input current to sensory neuron OLQVL for legs in Region D, only motor neuron RMDVR is activated, corresponding to the extension motion of the legs in Region D. However, for Region C, the input current is stimulated on sensory neuron OLQVR; then, only motor neuron RMDVL is activated, which determines the shortening behavior of the corresponding legs in Region C. Thanks to the alternate changes in the length of each leg in the four regions, the robot could move in the required direction, meaning that the robot completed the self-locomotion task by following the correct four-region control mechanism. This demonstrates that the BNN model could effectively control the robot’s locomotion by integrating the characteristics of the circuits in the whole-brain model with the robot’s kinematic joint motion, which is essential for the robot’s ontological locomotion control. The results also indicate that the effective and accurate real-time control of the robot was realized by the BNN model, which holds significant promise for the prospective deployment of the BNN model in practical applications.

In summary, based on the experimental and simulation outcomes, the robot demonstrates intelligent behavior in navigating toward a target location through an exploration of its surroundings and employing the BNN model to regulate its self-locomotion. The whole-brain network model of *C. elegans* enables efficient robot movement control, combined with biological neural control characteristics such as specific control circuits and appropriate encoding and decoding processes. These methodological frameworks collectively advance the field of biomimetic robot control, particularly in the pursuit of emulating the bionic behaviors of multi-legged robots through the utilization of biological neural network models.

## 4. Discussion

In this study, we established a whole-brain network model of *C. elegans* and designed the BNN control method to illustrate the BNN’s control abilities and dynamic characteristics and principles, before applying the BNN model to the robot control system in a closed loop. In the *C. elegans’* whole-brain neural network, sensory neurons activate certain motor neurons in discovered functional circuits, which also serve as the control center for the robot. Various circuits were utilized to control the robot using different control targets or steps by combining specific mechanisms and considering particular movement features of the robot. The electric responses of motor neurons were recorded and encoded into necessary variables, including digital instructions for the robot to move in a particular direction or continuous joint movement variables. The physical sensing or proprioception information of the robot, such as the distance to the ‘food’ or the leg region, is encoded into the input of the BNN model. Then, the BNN control system connects to the numeric simulation platform of the robot through the designed encoding and decoding methods. The method utilizes both the control characteristics of the whole-brain network of *C. elegans* and the locomotion properties of the robot, allowing for a more in-depth syncretic robotics control based on the biological neural network, with high-performing intelligence and completeness.

As a conceptual study, the results exhibit the efficiency of the BNN in controlling the intelligent behaviors of a robot. The behavior pattern relies on specific network configurations and functional circuits. By adopting the circuits of BNN directly (no training required), simple movement control of the robot can be realized on our numerical platform and then transferred to the experimental platform. By applying the whole-brain biological neural network method proposed in this study, the robot can realize autonomous motion toward an unknown target point, utilizing only a single local sensor. This is because the biological control mechanism enables the robot to explore its environment purely based on local experiences rather than absolute location coordinates. Consequently, the research indicates that the BNN possesses the potential to improve the robot’s mobility-based intelligence. As the preliminary research, this paper demonstrates a way to control a multi-legged robot using the BNN model, which is implemented on a virtual platform, and verified using an experimental platform. 

### 4.1. Contributions and Limitations

Here, we present a systematic and innovative methodology for applying the whole-brain model of the biological neural network to robotics control through the biological intelligence control mechanisms. Our research makes two main contributions. Firstly, we developed a real-time whole-brain network model that simulates the computational process occurring in biological neural activities, incorporating the HH model and synapse transmission based on a theoretical modelling. The simulation model serves as an integral platform for visualizing neuronal electrical activities and analyzing the dynamic implementation principles, including specific control circuits and their dynamic characteristics, according to the different behaviors exhibited by *C. elegans*. Additionally, the whole simulation model was open-sourced to facilitate future exploration and allow for its extension beyond the microscopic level to generate a more precise and comprehensive simulation. Secondly, leveraging the intelligence control capabilities inherent in neural networks, we demonstrated and validated the intelligent performance of the *C. elegans’* whole-brain neural network based on the real scientific robot control simulations and experiments. Our simulation platform of the whole-brain model was integrated with the robot control via the designed mechanisms and corresponding encoding and decoding methods. This aspect of this study introduces a novel and appropriate system for utilizing the biological whole-brain model to confer its intelligent control capabilities to robots, thereby offering a rational validation of the feasibility of using the BNN model to control robotics and generate intelligent behaviors, in contrast to previous methodologies such as PID control and artificial neural network methods.

The meaning of studying the whole-brain model of a creature is profound, not only in neuroscience but also in other areas of research, focusing on the intelligent implementation of the BNN mechanisms that control complicated behaviors [52]. Therefore, this study not only builds a whole-brain model but concentrates on how to combine the unique control methodology of the neural model with robotics to provide guidelines on bio-mimic intelligent control based on robotics. In the future, neuroscience and artificial intelligence could be two interdisciplinary subjects that are beneficial to each other, establishing a more efficient, advanced, agile, and robust control system for the realization of autonomous agents’ intelligence [53]. BNN control could achieve a better performance when applied to scientific robot control in future studies.

However, there are a few limitations of this study. In a BNN, the training and learning of the network occur continuously during the creature’s natural activities, involving synapse connection weight changes and the synapse connecting and breaking [54]. The detailed learning rules in the BNN are complicated and difficult to realize, so the research did not consider the learning process of the network. Because the BNN network changes remain relatively stable at different growth stages [54], the research established an intact biological whole-brain neural model referring to the existing data, which also ensures the accuracy of the model. We designed a closed-loop bio-mimic robot control for this model, ignoring the learning simulation of the network model. In future studies, the learning methods of the BNN model and the limitations of combining the BNN control system with the robot at a deeper level will be studied to improve the integration of the BNN-inspired intelligent control. Systematic methods of searching efficient control circuits in a biological neural network should also be studied in the future.

### 4.2. Analysis of Results

We aimed to achieve two goals: orientation control, causing the robot to move to a target point, and self-locomotion control, which is the robot joint motion control. Both the movement outcomes were satisfied using the BNN control inspired by *C. elegans*’ behaviors. For the control mission of moving to a target point, the most frequently used method is path planning built on the ANN control. However, according to the experiment conducted by the team, it is important to train the artificial neural network for a much longer time, using the network’s structural parameters and variables, to ensure an excellent performance. However, for the whole-brain neural network, the specified control circuits are especially important in highly efficient robot orientating control. While in some circumstances, the precision of the BNN control is lower than that of the well-trained ANN control, the exploration and adaptivity is much higher, which means the whole-brain bio-mimic control is completely referential. In an unknown environment, which requires the robot to have good self-adaption and learning abilities as it lacks navigation and sensor signals, the BNN model could help the robot reach a target through step-by-step exploration, similarly to *C. elegans*’ foraging behavior. 

Moreover, the BNN control not only helps the robot to fulfil intelligent control tasks, it also enables self-locomotion control. This signifies that when applied to an autonomous agent such as a robot, BNNs also possess self-motion abilities that are similar to those of *C. elegans*. Compared with the robot’s inverse kinetics control, the BNN control allows the robot to move consistently with the given instructions, and its performance was equivalent to that achieved using the current means of control. Although, at present, the BNN cannot control the robot by directly connecting external stimuli or directions to the joint motion using a one-time computation of circuits, the simulation and experimental results of this study demonstrate that the BNN possesses the ability to control a scientific robot through rational encoding and decoding. In future, no matter which features the robot possesses, the whole-brain neural network model could be combined more perfectly with the robot control in a closed loop to generate more intelligent and bionic behaviors in an efficient way. Therefore, it is significant to apply a whole-brain biological neural model to robotic control to observe its unique performance and demonstrate the BNN’s control ability, instead of using conventional control methods.

## Figures and Tables

**Table 1:** The parameter values in the HH model that are constants for every single neuron in the whole-brain model of *C. elegans*.

Parameters	Values
$C_{m}$	3.1 $\mu F/{\mathrm{cm}}^{2}$
${\bar{g}}_{l}$	0.289 nS
$V_{K}$	−75 mV
$V_{Na}$	55 mV
$V_{L}$	−84 mV
$V_{Ca}$	45 mV

**Table 2:** A summary of the circuits found in the whole-brain neural network of *C. elegans*, including corresponding sensory neurons, motor neurons, and the current stimuli range for activating motor neurons. The third column illustrates the activation states of the motor neurons caused by stimulation of the current in the corresponding sensory neuron. In terms of the oscillation behavior, the voltage response of motor neurons will generate oscillations, meaning that the spiking frequency will increase when the current acting on the sensory neuron increases within the value range. However, if there is only a single spiking behavior, the motor neurons will generate only one action potential when the current of the sensory neuron is larger than the threshold. The value range within which the input current will activate motor neurons is exactly calculated for a given group of parameters in this study for encoding and decoding. Most of the sensory neurons and motor neurons are both investigated and verified [22,24]. The relevant data and information for the neurons can be found at WormAtlas [24].

Sensory Neuron (Mechanosensory)	Motor Neuron	Current Stimuli Value Range (Unit: nA)
ASHL	DA, DB, VA, VB, VD, SMB, SMD, RMB, RMD	60- (One action potential only)
ADEL	DA, DB, VA, VB, VD	59–64 (Oscillation Range)
ADER	DA, DB, VA, VB, VD	59–68 (Oscillation Range)
CEPDL	DA, DB, VA, VB, VD, SMB, SMD, RMB, RMD	64–111 (Oscillation Range)
CEPDR	DA, DB, VA, VB, VD, SMB, SMD, RMB, RMD	64–91 (Oscillation Range)
CEPVL	DA, DB, VA, VB, VD, SMB, SMD, RMB, RMD	64–111 (Oscillation Range)
CEPVR	DA, DB, VA, VB, VD, SMB, SMD, RMB, RMD	64–85 (Oscillation Range)
PDEL	DA, DB, VA, VB, SMDVL	60–190 (Oscillation Range)
PDER	DA, DB, VA, VB, VD, SMB, SMD, RMB, RMD	60–88 (Oscillation Range)
OLQDL	RMDDR	91– (One action potential only)
OLQDR	RMDDL	91– (One action potential only)
OLQVL	RMDVR	91– (One action potential only)
OLQVR	RMDVL	94– (One action potential only)

## Data Availability

The data for this study of whole-brain neural network building is shared through the link: https://github.com/HuKangxin/C.-elegans-whole-brain-neural-network-building-and-simulation/tree/main/C-elegans, accessed on 20 October 2023.

## Supplementary Materials

The following supporting information can be downloaded at: https://www.mdpi.com/article/10.3390/biomimetics9060314/s1.
