# Supplementary material for: Innate Orientating Behavior of a Multi-Legged Robot Driven by the Neural Circuits of C. elegans
Source: Biomimetics (Basel). 2024 May 23;9(6):314. doi: 10.3390/biomimetics9060314 (PMC11201571; doi:10.3390/biomimetics9060314)
Supplement: Supplementary file 1 [file biomimetics-09-00314-s001.zip › Tables/Table S1.pdf]

**Type of Ion Channel Distributed on Neurons**	**Gene Expression**
Sodium	MEC-10
Calcium	TRP-4
	ITR-1
	OSM-9
Potassium	SLO-2
	SLO-1
	SHL-1
	SHK-1
	NCA-2
	KVS-1
	KQT-3
	KCNL-4
	KCNL-1
	IRK-3
	IRK-2
	EGL-36
	IRK-1
