# Supplementary material for: Innate Orientating Behavior of a Multi-Legged Robot Driven by the Neural Circuits of C. elegans
Source: Biomimetics (Basel). 2024 May 23;9(6):314. doi: 10.3390/biomimetics9060314 (PMC11201571; doi:10.3390/biomimetics9060314)
Supplement: Supplementary file 1 [file biomimetics-09-00314-s001.zip › Tables/Table S2.pdf]

**Parameters**	**Values**
Total Weight	1.819 *kg*
Moment of Inertia	0.029 $kg \bullet m^2$
Maximum Radius	406 *mm*
Minimum Radius	206 *mm*
Limit of Stretching Velocity of Legs	26 *mm/s*
Foot Radius	32 *mm*
