# Supplementary figures and images for: Innate Orientating Behavior of a Multi-Legged Robot Driven by the Neural Circuits of C. elegans
Source: Biomimetics (Basel). 2024 May 23;9(6):314. doi: 10.3390/biomimetics9060314 (PMC11201571; doi:10.3390/biomimetics9060314)

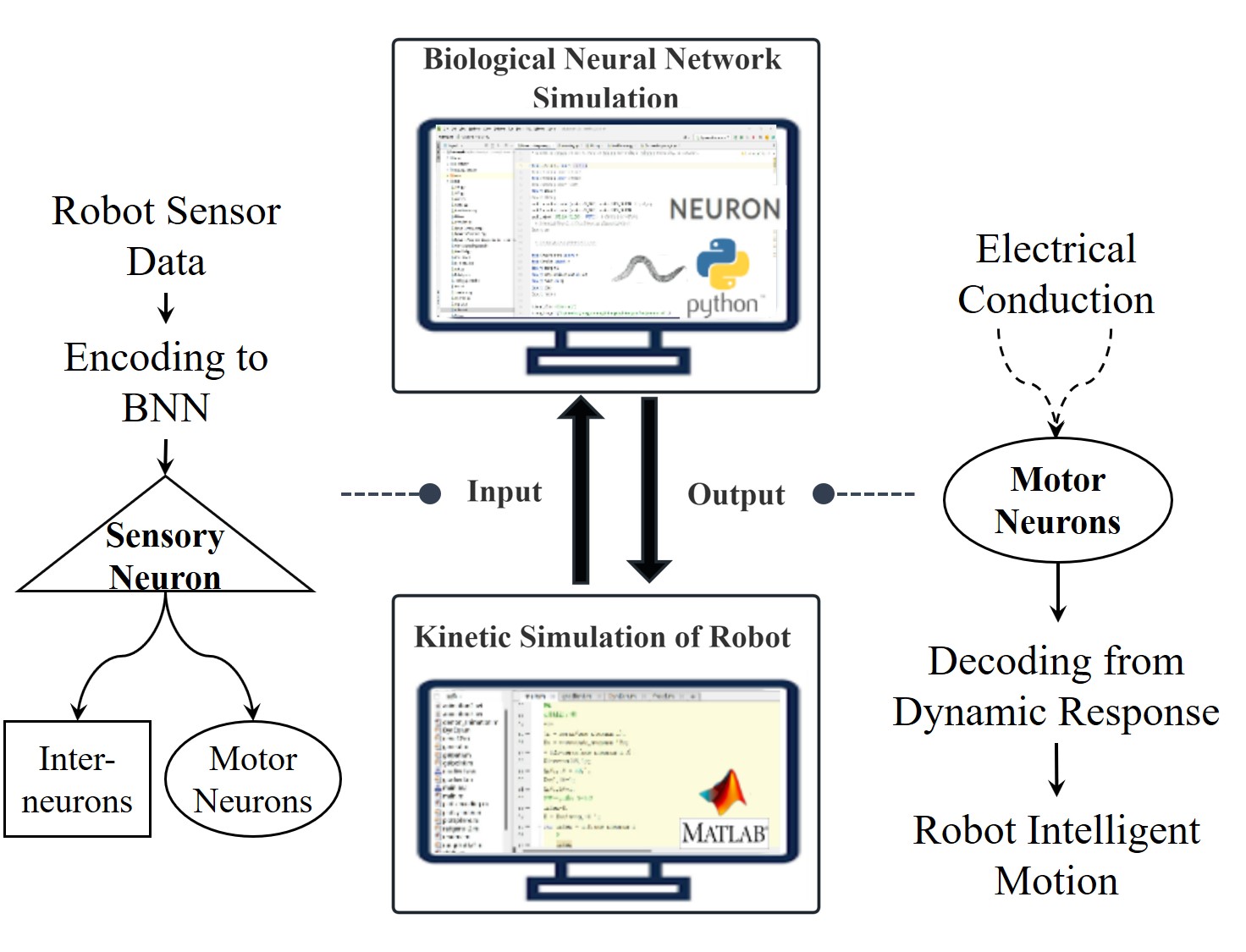

Supplement: Supplementary file 1 [file biomimetics-09-00314-s001.zip › Pictures/Figure S1.jpg]

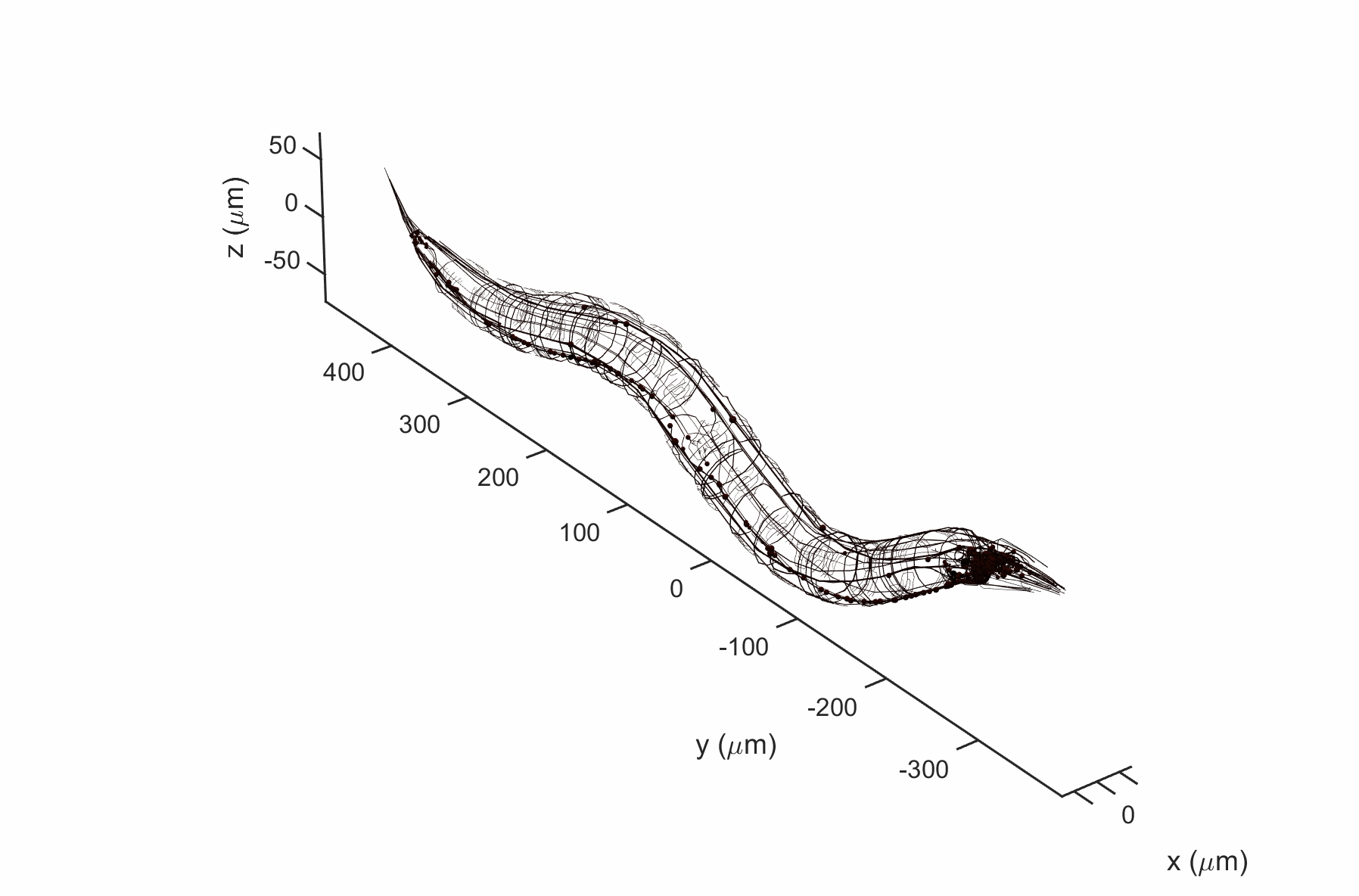

Supplement: Supplementary file 1 [file biomimetics-09-00314-s001.zip › Pictures/Figure S2.gif]

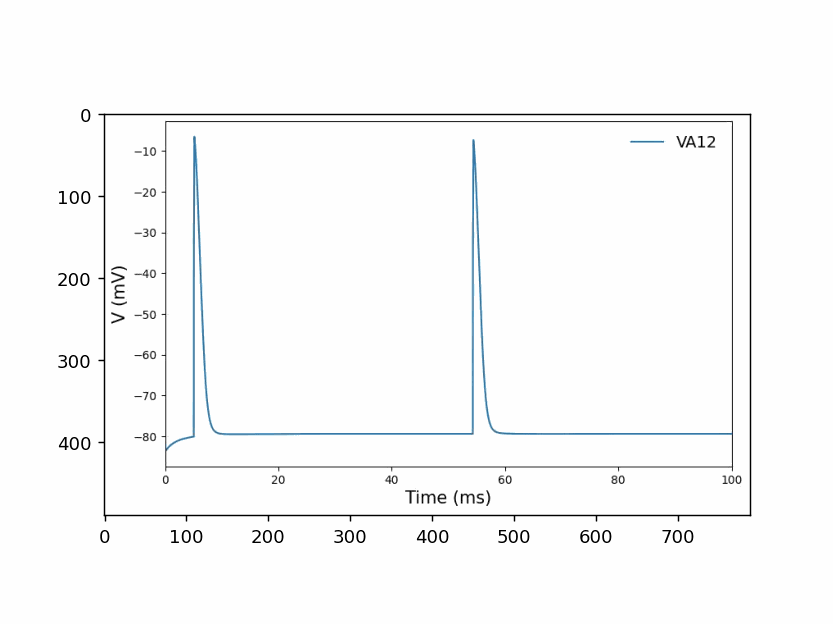

Supplement: Supplementary file 1 [file biomimetics-09-00314-s001.zip › Videos/Video S1.gif]
